# Could BMMNCs therapy reduce the mid- and long-term rate of total hip arthroplasty of femoral head necrosis?: A systematic review and meta-analysis

**DOI:** 10.1097/MD.0000000000034311

**Published:** 2023-07-28

**Authors:** Xiao Chen, Jing Chen, Yanji Duan, Chang Chen, Yuan Cao

**Affiliations:** a Department of Orthopedic Surgery, The First People’s Hospital of Neijiang, Neijiang, China; b Department of Neonatology, The First People’s Hospital of Neijiang, Neijiang, China.

**Keywords:** arthroplasty, bone marrow mononuclear cells, meta-analysis, osteonecrosis of the femoral head, therapy

## Abstract

Osteonecrosis of the femoral head (ONFH) is a cause of hip pain and early joint lesion in patient. The hip-preserving treatments are especially important for patients in early stage of ONFH. However, it is controversial of the effectiveness and safety of bone marrow mononuclear cells (BMMNCs) in the treatment of ONFH. The aim of the study was to explore the mid- and long-term efficacy (particularly the rate of total hip arthroplasty [THA]) with BMMNCs in treatment of ONFH.

**Methods:**

PubMed, Web of Science, Embase, OVID, Cochrane Libriary, CNKI, and Google Scholar databases were searched for relevant randomized controlled trials or non-randomized controlled trials from inception to October 15, 2022. Methodological quality of the trials was assessed, relevant data were extracted, and RevMan 5.3 and Stata 15.0 software were used to perform the meta-analysis of parameters related to the consequences.

**Results:**

A total of 22 articles were included, including 1923 patients. Meta-analysis results showed that the treatment of BMMNCs has a significantly lower incidence of THA (odds ratio [OR] = 0.33; 95% confidence interval [CI] = 0.27–0.41, *P* < .00001), radiographic progression rate (OR = 0.37; 95% CI = 0.21–0.63, *P* = .0003) and visual analog score at 24 months (mean difference [MD] = −11.84; 95% CI = −14.86 to −8.82, *P* < .00001), and has higher Harris hip score (MD = 6.90; 95% CI = 4.56–9.24, *P* < .00001), improvement of visual analog score at 24 months (MD = 6.87; 95% CI = 1.84–11.89, *P* = .007) and Merle D’Aubigne and Postel hip score (MD = 0.79; 95% CI = 0.14–1.44, *P* = .02). But there was no significant difference in the Western Ontario and McMaster University Osteoarthritis index (MD = −6.32; 95% CI = −16.76 to 4.12, *P* = .24) and incidence of complication (OR = 0.86; 95% CI = 0.52–1.42, *P* = .56).

**Conclusion:**

Current evidence supports that BMMNCs therapy could reduce the mid- and long-term rate of THA, improve hip function, alleviated the degree of hip pain, delay the progression of imaging staging and not increase the rate of complication, which maybe serve as a preferred option for treating ONFH.

## 1. Introduction

Osteonecrosis of the femoral head (ONFH) is a cause of hip pain and early joint lesion in patient and is a progressive pathological process, usually caused by disruption of the blood supply to the femoral head and elevation of intraosseous pressure.^[1,2]^ It mostly affects young adults, causing considerable morbidity.^[3]^ The annual incidence of ONFH in the USA is estimated to be 10,000 to 20,000 cases.^[4]^ The main causes of ONFH include trauma, alcohol abuse, the use of a large number of corticosteroids, organ transplantation, some inflammatory or autoimmune diseases, sickle cell anemia, lipid metabolism disorder, viral infection and so on.^[5–7]^ Multiple risk factors lead to the destruction of microcirculation of the femoral head and local necrosis.^[8]^

The disease usually progresses to femoral head collapse and secondary symptomatic hip arthritis. In most patients without effective early treatment, this type of osteonecrosis can develop into femoral head collapse with subsequent hip joint destruction and eventually require total hip arthroplasty (THA) to restore joint function.^[9–11]^ This condition usually affects the young patients. However, there are many complications after THA, the service life of the artificial hip joint is limited, and the possibility of needing revision is high, which brings a huge burden to patients,^[12]^ and it cannot be expected to increase the patient’s lifetime, so the hip-preserving treatments are especially important for these patients in early stage of ONFH.^[13]^

At present, the main treatment methods of joint preservation procedure include bisphosphonate, vasodilatation and other drugs, core decompression (CD), femoral osteotomy, bone transplantation, biomaterial implantation and so on.^[14]^ However, these treatments are difficult to regenerate the necrotic bone tissue and prevent the progression of femoral head necrosis. Therefore, it is very important to find a way to prevent the progression of osteonecrosis of femoral head and effectively repair the osteonecrosis of femoral head. In recent years, several authors have reported encouraging outcomes of adjuvant regenerative therapies when combined with CD for treatment of ONFH. In these procedures, bone marrow stem cells, bone marrow mononuclear cells (BMMNCs), bone marrow aspiration concentrate, platelet rich plasma, and other growth factors (i.e., bone morphogenetic proteins, angiogenic growth factors, interleukins, and cytokines) are implanted into the necrotic region after CD to stimulate osteogenesis and improve healing. BMMNCs are thought to be one of the most useful for angiogenesis and bone formation.^[15–18]^ Early studies have shown that BMMNCs therapy for ONFH can reduce pain, improve hip function, and delay disease progression.^[19]^ However, some studies have shown that the efficacy of mononuclear cells in the treatment of ONFH is not significant, and its efficacy is affected by many factors such as cell source, dose, cell frequency, patient stage and so on.^[20]^

To date, there have been a few meta-analyses and systematic reviews of adjuvant regenerative therapy in the treatment of ONFH, most of these reports bone marrow stem cells^[21–26]^ but not BMMNCs for ONFH, owns few included studies, fails to analyze the changes in the volume of the necrotic lesion, lacks studies on the long-term efficacy after cell transplantation and the efficacy comparison of different doses. Which increasing the risks for biases (i.e., sample, selection, and collider) and error, and makes the safety and effectiveness of BMMNCs in the treatment of ONFH controversial.

Therefore, we conduct a meta-analysis to investigate the long-term efficacy after cell transplantation and the efficacy comparison of different doses and safety of BMMNCs in the treatment of ONFH. In this study, it is hypothesized that BMMNCs therapy could improve hip function, alleviated the degree of hip pain, delay the progression of imaging staging, reduce the femoral head necrosis volume and reduce the rate of total hip arthroplasty, and not increase the rate of complication between 2 groups.

## 2. Materials and methods

This systematic review and meta-analysis was conducted according to the recommendations of the Cochrane Collaboration and is reported per Preferred Reporting Items for Systematic Reviews and Meta-Analysis guidelines.

### 2.1. Data sources and searches

PubMed, Web of Science, Embase, OVID, Cochrane Libriary, CNKI, and Google Scholar databases were searched for relevant randomized controlled trials or non-randomized controlled trials from inception to October 15, 2022. No limits on publication date or language were applied to the databases searched. The following Mesh terms, keywords, and phrase were used to search the databases: “femur head necrosis,” “osteonecrosis of femoral head,” “femoral head necrosis,” “femur head necroses,” “aseptic necrosis of femur head,” “ischemic necrosis of femoral head,” “avascular necrosis of femur head,” “ONFH,” “ANFH,” “mononuclear cell,” “bone marrow mononuclear cell,” “mononuclear bone marrow cell,” “peripheral blood monouclear cells,” “bone marrow concentrate,” “bone marrow aspirate concentrate,” “stem cell,” “stromal cell,” “MNCs,” “PBMNCs,” and “BMMNCs.” Additional searching of the potentially relevant literature included a focused search of Google Scholar as well as Clinicaltrials.gov. Search results were exported to Endnote X7 where duplicates were screened and removed before initial review of titles and abstracts.

### 2.2. Inclusion and exclusion criteria

Studies were selected based on the following inclusion criteria: study design: randomized controlled trials (RCTs) or non-RCTs such as cohort studies, case control studies, etc.; patients: patients diagnosed with osteonecrosis of femoral head over than 18 year-old; interventions: bone marrow mononuclear cells versus other methods with or without implants (such as autologous bone graft, angioconductive bioceramic rod graft, β-tricalcium phosphate, porous hydroxyapatite, porous tantalum rod, interconnected porous hydroxyapatite and so on); outcomes: primary outcomes: THA conversion rate and Harris hip score (HHS); second outcomes: the Western Ontario and McMaster University Osteoarthritis (WOMAC) index, Lequesne index, Merle D’Aubigne and Postel hip score, visual analog scale (VAS), radiographic progression rate (or collapse rate), femoral head necrosis volume and complication.

Exclusion criteria were study objective or intervention measures failed to meet the inclusion criteria; protocols, case reports, animal studies, human autopsy and other non-clinical trials, reviews, conference papers and studies without use-able data; and repeatedly published literature and duplicate data from another study.

### 2.3. Data extraction and quality evaluation

Two authors searched, screened and extraction the data from all eligible article independently, and any disagreements were resolved by discussion and consensus among the authors. Studies were then selected by reading the title, abstract and full text. If necessary, attempts were made to contact the authors for required data.

The following information was extracted from all eligible articles: general information (name of first author, year of publication, region where the population resided, type of study, sample size, mean ages, and interventions) and outcomes (as defined above).

The evaluation criteria and methods of the included trials was followed the Cochrane Collaboration proposal. For non-randomized controlled trials, the Newcastle-Ottawa Scale was used for bias assessment. For randomized controlled trials, appraisal criteria included random sequence generation, allocation concealment, blinding of participants and personnel, blinding of outcome assessment, incomplete outcome data, selective reporting, and other sources of bias. Each of these factors was recorded as low risk, unclear risk, or high risk.

### 2.4. Statistical analysis

The extracted data were pooled using Review Manager 5.3. The mean difference (MD) and odds ratio (OR) were introduced to evaluate the differences among interventions on continuous variables and dichotomous variables, respectively, and 95% confidence interval (CI) was calculated as well to examine the significance. Heterogeneity of the included studies was evaluated using Higgins *I*^2^. A random-effect model was used when apparent heterogeneity was detected (*I*^2^ ≥ 50% or *P* < .05). Otherwise, a fixed effect model was used (*I*^2^ < 50% or *P* ≥ .05). Potential publication bias was judged by Begg’s and Egger’s tests. Sensitivity analysis was performed to evaluate the robustness of the combined data. A *P* value < .05 was regarded as statistically significant for all tests.

## 3. Results

### 3.1. Search results

**Figure 1.**
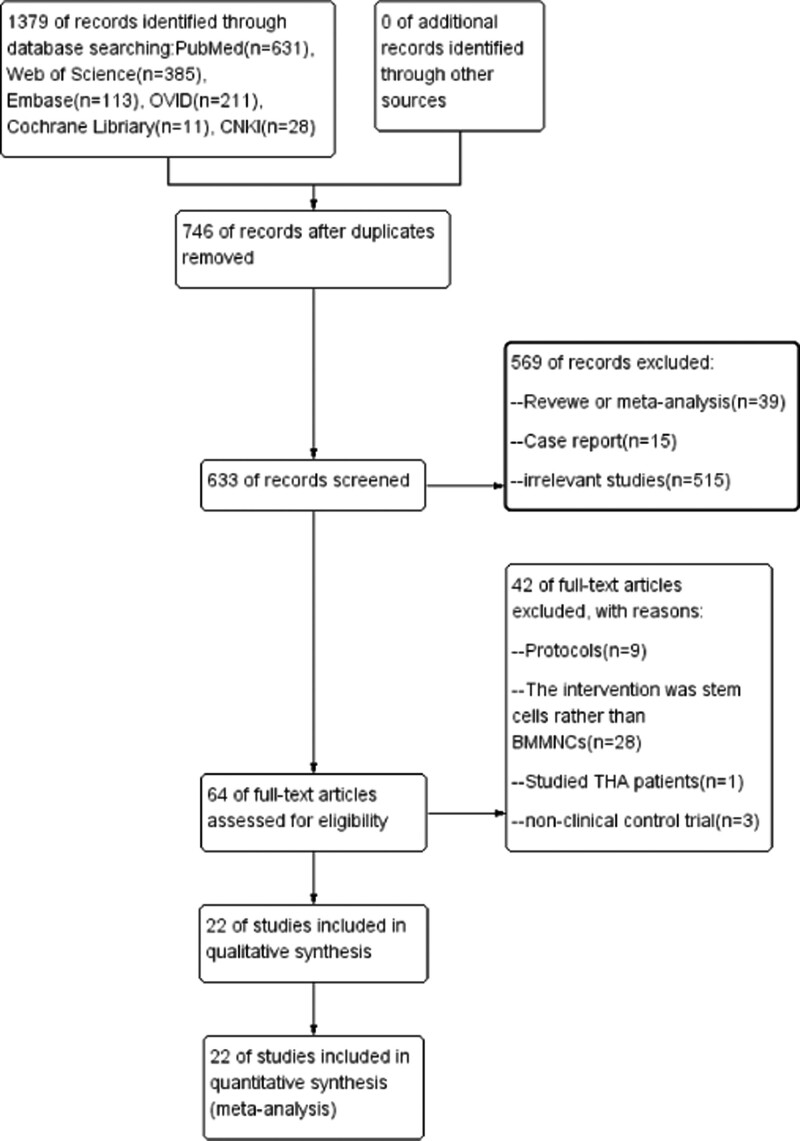
Flow chart of study identification and selection.

In accordance with the search strategy, 1379 publications were identified from electronic medical databases. In addition, the title list was imported into Endnote software, 1315 studies which was duplicated or did not meet the inclusion criteria such as reviews, meta-analysis, animal-based studies were excluded. We included RCTs and non-RCTs because we required sufficient data to accomplish a good meta-analysis study. When only RCT articles were included, the number of references did not support the analysis. After reading the full text, 22^[5,13,27–46]^ articles were finally included and used to provide primary data for further analysis, there were 10 randomized controlled trials,^[30–32,34,37,38,40–43]^ 12 case-control studies,^[5,13,27–29,33,35,36,39,44–46]^ 20 English literatures,^[5,13,27–35,37–45]^ and 2 Chinese literatures,^[36,46]^ including 1923 patients. The study selection process and reasons for exclusion are summarized in Figure 1. And the main characteristics of these studies and patients are summarized in Table 1.

**Table 1:** Summary of study and patient characteristics.

Author and year	Country	Intervention	Cell count	No. of hips	Age (yr)	Follow-up time (mo)	Outcome
Bootanapibul 2021	America	CD+BMMNCs CD	Not given	50 33	38 ± 13 43 ± 10	36 ± 23	(6)(8)
Gangji 2004	Belgium	CD+BMMNCs CD	(2 ± 0.3)*10^9^	10 8	40.9 ± 9.8 48.8 ± 11.2	24	(2)(3)(5)(6)(7)(8)(9)
Gangji 2011	Belgium	CD+BMMNCs CD	(1.9 ± 0.2)*10^9^	13 11	42.2 ± 2.6 45.7 ± 2.8	60	(3)(5)(6)(7)(8)(9)
Hauzeur 2018	Belgium	CD+BMMNCs CD	(3.46 ± 0.36)*10^9^	23 23	48.0 ± 2.8 49.7 ± 3.2	24	(5)(6)(8)(9)
Hauzeur 2020	Belgium	BMMNCs OC	(3.4 ± 3.0)*10^9^	26 27	50 ± 12 51 ± 10	36	(2)(3)(5)(6)(8)(9)
Hernigou 2018	France	CD+BMMNCs CD	(5.16 ± 1.6)*10^8^	125 125	36 (18–54) 36 (18–54)	360	(1)(2)(5)(6)(8)
Kang 2018	South Korea	CD+BMMNCs CD	(2.10 ± 2.07)*10^8^	53 53	46.0 ± 9.3 47.3 ± 9.7	51.36	(6)(8)(9)
Li 2021	China	CD+BMMNCs+ABG CD+ABR+β-TCR	Not given	22 29	35.4 ± 11.1 39.4 ± 10.4	24	(1)(8)(9)
Liu 2013	China	CD+BMMNCs+PH CD+PH	(1.57 ± 0.24)*10^8^	26 27	38.0 ± 4.9 38.1 ± 6.1	26.7 ± 8.0 24.9 ± 4.5	(1)(5)(6)(8)(9)(10)
Ma 2014	China	CD+BMMNCs+ABG CD+ABG	3*10^9^	25 24	35.6 ± 8.05 34.78 ± 11.48	24	(2)(3)(5)(6)(8)(9)
Mao 2015	China	BMMNCs+PTR PTR	(2.47 ± 0.5)*10^8^	48 41	34.60 ± 11.50 36.12 ± 11.34	36	(1)(6)(8)(9)
Nally 2017	Italy	CD+BMMNCs CD	Not given	16 47	41 (25–63) 38 (21–63)	72	(8)
Pepke 2016	Germany	CD+BMMNCs CD	(1.19 ± 0.15)*10^9^	11 14	44.3 ± 3.4 44.5 ± 3.3	24	(1)(5)(7)(8)
Pilge 2016	Germany	CD+BMMNCs CD	Not given	10 10	38.3 ± 13.18 38.4 ± 13.47	4–69	(4)(8)
Sen 2012	India	CD+BMMNCs CD	5*10^8^	26 25	Not given	24	(1)(9)
Yamasaki 2010	Japan	CD+BMMNCs+IP-CHA CD+IP-CHA	1*10^9^	30 9	41 (18–64) 49 (28–73)	31	(6)(8)
Tabatabaee 2015	Iran	CD+BMMNCs CD	Not given	14 14	31 ± 11.4 26.8 ± 5.8	24	(2)(5)(6)(8)
Cruz-Pardos 2016	Spain	CD+BMMNCs CD	Not given	41 19	42.56 (23–70) 36.74 (20–68)	64	(4)(6)(8)(9)
Zhang 2019	China	CD+BMMNCs CD	Not given	44 44	34.9 ± 7.2 34.9 ± 7.2	54.0 ± 33.1 50.5 ± 34.2	(1)(5)(8)(9)
Liu 2013	China	CD+BMMNCs+PH CD+PH	(1.57 ± 0.24)*10^8^	57 27	39.4 ± 7.2 38.0 ± 6.0	28.0 ± 8.6 24.9 ± 4.5	(1)(5)(8)(9)
Tomaru 2022	Japan	BMMNCs Observation alone	Not given	387 171	40.1 (14.3–77.2) 48.9 (14.3–84.4)	72	(6)(8)
Hoogervorst 2022	America	CD+BMMNCs CD	Not given	61 24	33.1 ± 10.0 39.8 ± 12.6	60	(6)(8)

ABG = autologous bone graft, ABR = angioconductive bioceramic rod grafting, BMMNCs = bone marrow mononuclear cells, CD = core decompression, IP-CHA = interconnected porous hydroxyapatite, OC = osteoblastic cell, PH = porous hydroxyapatite, PTR = porous tantalum rod, β-TCR = β-tricalcium phosphate. Outcome: (1) Harris hip score; (2) the Western Ontario and McMaster University Osteoarthritis index; (3) Lequesne index; (4) Merle D’Aubigne and Postel hip score; (5) visual analog score; (6) radiographic progression rate; (7) femoral head necrosis volume; (8) total hip arthroplasty conversion rate; and (9) complication.

### 3.2. Quality assessment and basic information

**Figure 2.**
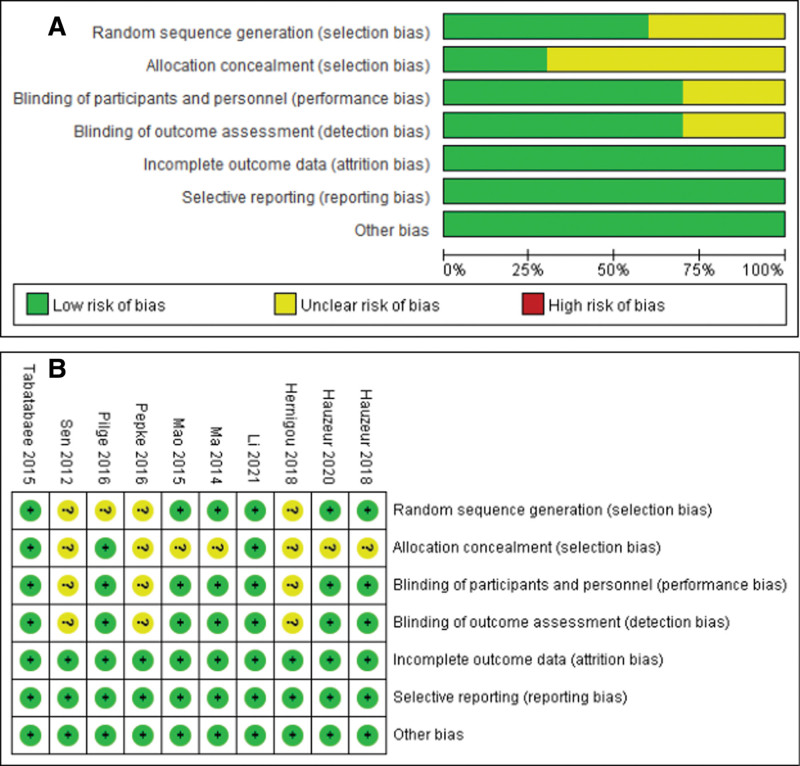
Risk of included studies. (A) Risk of bias graph. (B) Risk of bias summary.

The quality of the included RCTs was assessed using the Cochrane Collaboration’s “Risk of bias.” The risk of bias assessment of included studies is given in Figure 2. The risk of bias of the included non-RCTs evaluated with the Newcastle-Ottawa Scale score (the score ≥ 7 indicated good literature quality) is demonstrated in Table 2.

**Table 2:** The Newcastle-Ottawa Scale score of non-RCT.

Study	1	2	3	4	5	6	7	8	Score
Bootanapibul 2021	1	1	0	1	2	1	1	1	8
Gangji 2004	1	1	0	1	2	1	1	1	8
Gangji 2011	1	1	0	1	2	1	1	1	8
Kang 2018	1	1	0	1	1	1	1	1	7
Liu 2013	1	1	0	1	1	1	1	1	7
Nally 2017	1	1	0	1	2	1	1	1	8
Yamasaki 2010	1	1	0	1	2	1	1	1	8
Cruz-Pardos 2016	1	1	0	1	2	1	1	1	7
Zhang 2019	1	1	0	1	1	1	1	1	7
Liu 2013	1	1	0	1	2	1	1	1	8
Tomaru 2022	1	1	0	1	2	1	1	1	8
Hoogervorst 2022	1	1	0	1	2	1	1	1	8

First item: is the case definition adequate; second item: representativeness of the cases; third item: selections of controls; fourth item: definition of controls; fifth item: comparability of case and controls on the basis of the design or analysis; sixth item: ascertainment of exposure; seventh item: same method of ascertainment for cases and controls; and eighth item: non-response rate. each item is 1 point except item 5, which has a value of 2 points.

### 3.3. Meta-analysis of primary outcomes

#### 3.3.1. THA conversion rate.

**Figure 3.**
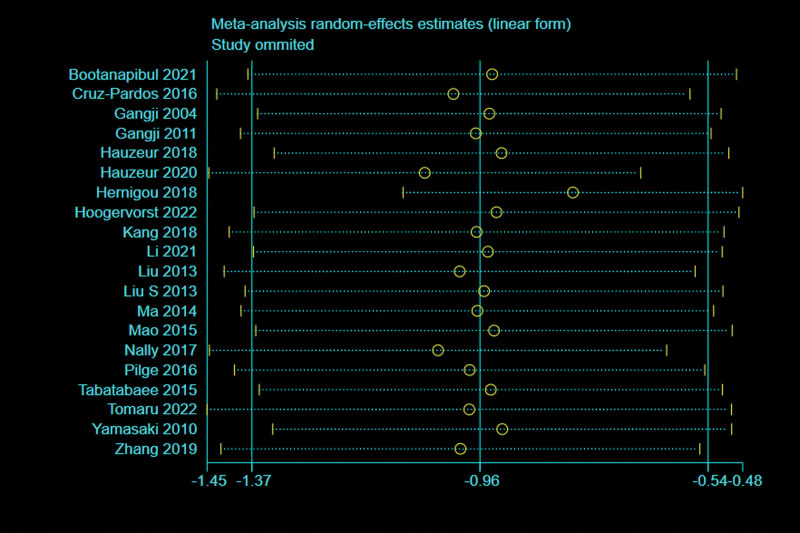
Sensitivity analysis of THA conversion rate. THA = total hip arthroplasty.

**Figure 4.**
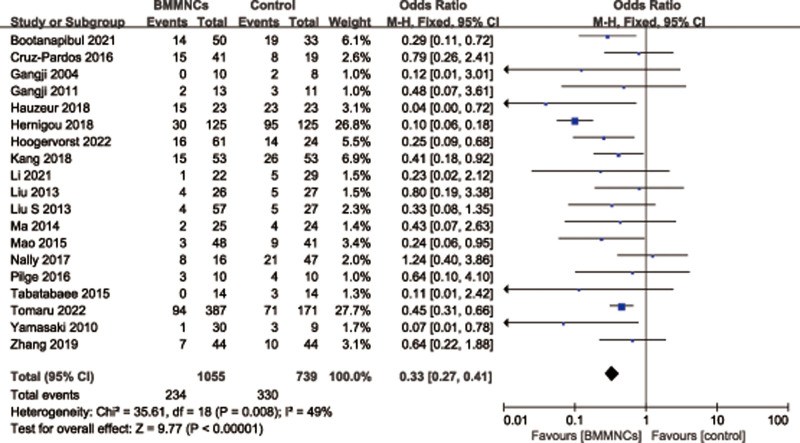
Forest plot of THA conversion rate. THA = total hip arthroplasty.

A total of 20 studies^[5,13,27–39,41,43–46]^ with 1847 patients reported the relevant data regarding the rate of THA. There was significant heterogeneity observed among articles (*I*^2^ = 58%, *P* = .0003), therefore, a random effects model was used. Data pooling revealed a significantly lower THA conversion rate in BMMNCs group compared with control group (OR = 0.39; 95% CI = 0.25–0.59, *P* < .0001). Considering the significant heterogeneity, the Stata software was used for further sensitivity analysis (Fig. 3), and the results showed that heterogeneity of THA conversion rate changed significantly after deleting the study of Hauzeur et al.^[31]^ Meta-analysis was conducted on the remaining 19 trials.^[5,13,27–30,32–39,41,43–46]^ A fixed effects model was used due to low heterogeneity (*P* = 49%, *P* = .008). Pooled data also showed that the rate of THA was significantly lower in the BMMNCs group (OR = 0.33; 95% CI = 0.27–0.41, *P* < .00001) (Fig. 4).

Subgroup analysis was performed according to different follow-up period. Among them, 8 trials^[29,30,34–37,43,45]^ reported the THA conversion rate with follow-up time at 12 to 24 months, and the results showed that the difference of the incidence of THA between 2 groups was statistically significant (OR = 0.64; 95% CI = 0.13–0.49, *P* < .0001), and 12 trials^[5,13,27,28,31–33,38,39,41,44,46]^ reported the THA conversion rate with follow-up time more than 36 months, it also showed that the difference of the rate of THA between 2 groups was statistically significant (OR = 0.45; 95% CI = 0.27–0.75, *P* = .002). Subgroup analysis was performed according to different therapy dose of BMMNCs. Among them, 5 trials^[32,33,35,36,38]^ reported the THA conversion rate with cell therapy dose unit is 10^8^, and the results showed that the difference of the incidence of THA between 2 groups was statistically significant (OR = 0.27; 95% CI = 0.12–0.62, *P* = .002), and 6 trials^[28–31,37,45]^ reported the THA conversion rate with cell therapy dose unit is 10^9^, it showed that no statistically significant difference of the rate of THA between 2 groups (OR = 0.30; 95% CI = 0.07–0.31, *P* = .10). Subgroup analysis was performed according to the presence or absence of implants. Among them, 6 trials^[34–38,45]^ reported the THA conversion rate with implants, and the results showed that the difference of the incidence of THA between 2 groups was statistically significant (OR = 0.33; 95% CI = 0.17–0.64, *P* = .001), and 14 trials^[5,13,27–33,39,41,43,44,46]^ reported the THA conversion rate without implants, it also showed that the difference of the incidence of THA between 2 groups was statistically significant (OR = 0.41; 95% CI = 0.25–0.70, *P* = .001) (Table 3).

**Table 3:** Subgroup analysis of variety of outcomes.

Outcome	No. of study	No. of hips	Heterogeneity		Effects model	Results of meta-analysis	
			*P*	*I* ^2^		MD/OR (95% CI)	*P*
THA conversion rate							
FU = 12–24 mo	8	368	.53	0%	Fixed	0.64 (0.13, 0.49)	<.0001
FU ≥ 36 mo	12	1479	<.0001	72%	Random	0.45 (0.27, 0.75)	.002
Dose unit 10^8^	5	582	.01	68%	Random	0.27 (0.12, 0.62)	.002
Dose unit 10^9^	6	229	.02	63%	Random	0.33 (0.08, 1.36)	.12
With implants	6	365	.63	0%	Fixed	0.33 (0.17, 0.64)	.001
Without implants	14	1482	<.0001	69%	Random	0.41 (0.25, 0.70)	.001
Complication							
FU = 12–24 mo	3	155	.45	0%	Fixed	0.77 (0.31, 1.90)	.57
FU ≥ 36 mo	5	324	.48	36%	Fixed	1.02 (0.53, 1.96)	.95
Dose unit 10^8^	3	226	.97	0%	Fixed	0.56 (0.25, 1.26)	.16
Dose unit 10^9^	3	105	.51	0%	Fixed	2.28 (0.78, 6.64)	.13
With implants	3	226	.97	0%	Fixed	0.56 (0.25, 1.26)	.16
Without implants	5	253	.35	10%	Fixed	1.13 (0.59, 2.16)	.72
Radiographic progression rate							
FU = 12–24 mo	6	235	.06	54%	Random	0.20 (0.07, 0.61)	.004
FU ≥ 36 mo	9	1308	<.00001	81%	Random	0.46 (0.24, 0.88)	.02
Dose unit 10^8^	4	500	.003	79%	Random	0.27 (0.10, 0.71)	.008
Dose unit 10^9^	6	229	.0008	76%	Random	0.30 (0.07, 1.25)	.10
With implants	4	232	.65	0%	Fixed	0.15 (0.07, 0.31)	<.00001
Without implants	11	1311	<.00001	78%	Random	0.47 (0.25, 0.89)	.02
VAS							
Dose unit 10^8^	3	287	.02	75%	Random	−12.21 (−16.26, −8.17)	<.00001
Dose unit 10^9^	1	49	–	–	Fixed	−9.54 (−11.31, −7.77)	<.00001
With implants	3	186	.79	0%	Fixed	−9.66 (−11.23, −8.09)	<.00001
Without implants	3	386	.08	60%	Random	−14.63 (−17.21, −12.06)	<.00001
Hairs							
FU = 12–24 mo	3	188	.50	0%	Fixed	6.06 (4.23, 7.88)	<.00001
FU ≥ 36 mo	3	427	.04	70%	Random	7.41 (2.00, 12.82)	.007
Dose unit 10^8^	4	476	.11	50%	Random	7.37 (5.05, 9.69)	<.00001
With implants	4	277	.12	48%	Fixed	7.11 (5.58, 8.64)	<.00001
Without implants	2	338	.07	70%	Random	4.73 (0.26, 9.20)	.04

FU = follow-up, MD = mean difference, OR = odds ratio.

#### 3.3.2. Harris hip score.

**Figure 5.**
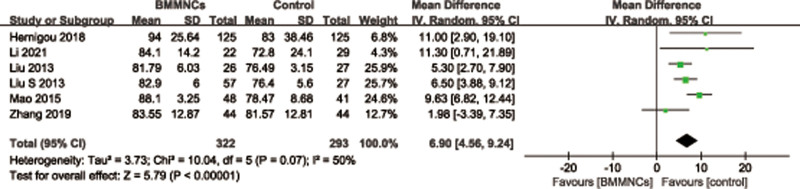
Forest plot of the HHS. HHS = Harris hip score.

**Figure 6.**
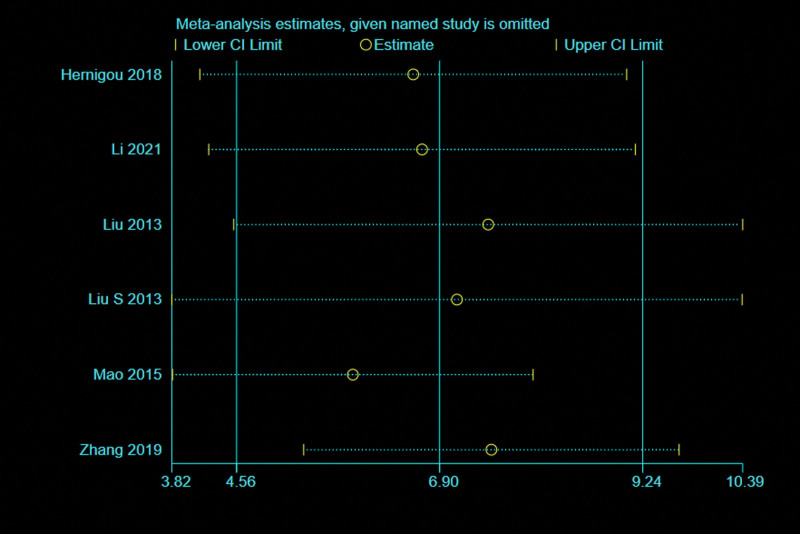
Sensitivity analysis of the HHS. HHS = Harris hip score.

Six trials^[32,34–36,38,46]^ with a total of 615 patients provided data on the HHS. A random effects model was used because of significant heterogeneity (*I*^2^ = 50%, *P* = .0.07). It suggested that the HHS was significantly higher in the BMMNCs group than the controlled group (MD = 6.90; 95% CI = 4.56–9.24, *P* < .00001) (Fig. 5). Considering the significant heterogeneity, the Stata software was used for further sensitivity analysis (Fig. 6), and the results showed that the total combined effect size of HHS didn’t change significantly after removing single study one by one, suggesting that the results were robust.

Subgroup analysis was performed according to different follow-up period. Among them, 3 trials^[34–36]^ reported the HHS with follow-up time at 12 to 24 months, and the results showed that the difference of the HHS between 2 groups was statistically significant (MD = 6.06; 95% CI = 4.23–7.88, *P* < .00001), and 3 trials^[32,38,46]^ reported the HHS with follow-up time more than 36 months, it also showed that the difference of the HHS between 2 groups was statistically significant (MD = 7.41; 95% CI = 2.00–12.82, *P* = .007). Four trials^[32,35,36,38]^ reported the HHS with cell therapy dose unit is 10^8^, and the results showed that the difference of the HHS between 2 groups was statistically significant (MD = 7.37; 95% CI = 5.05–9.69, *P* < .00001). Subgroup analysis was performed according to the presence or absence of implants. Among them, 4 trials^[34–36,38]^ reported the HHS with implants, and the results showed that the difference of the HHS between 2 groups was statistically significant (MD = 7.11; 95% CI = 5.58–8.64, *P* < .00001), and 2 trials^[32,46]^ reported the HHS without implants, it also showed that the difference of the HHS between 2 groups was statistically significant (MD = 4.73; 95% CI = 0.26–9.20, *P* = .04) (Table 3).

### 3.4. Meta-analysis of second outcomes

#### 3.4.1. Visual analog scale.

**Figure 7.**
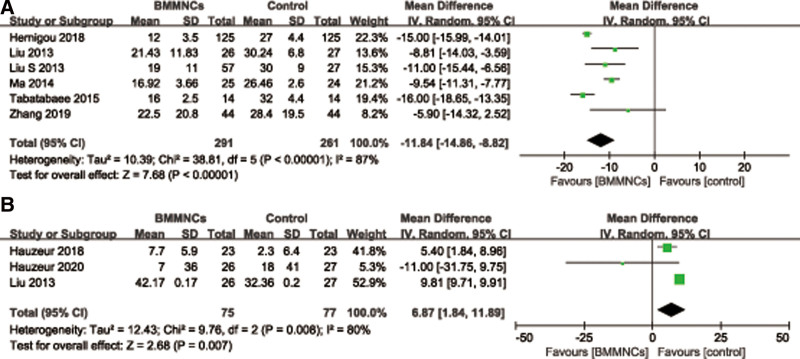
Forest plot of the VAS (A) and the improvement of VAS (B) at 24 months. VAS = visual analog score.

Six studies^[32,35–37,43,46]^ with 552 patients reported the relevant data regarding the VAS at 24 months. A random effects model was used because of high heterogeneity (*I*^2^ = 87%, *P* < .00001). And data pooling indicated that the VAS at 24 months was significantly lower in the BMMNCs group (MD = −11.84; 95% CI = −14.86 to −8.82, *P* < .00001) (Fig. 7A). Considering the significant heterogeneity, the Stata software was used for further sensitivity analysis, and the results showed that the total combined effect size of VAS didn’t change significantly after removing single study one by one, suggesting that the results were robust. Meanwhile, 3 trials^[30,31,35]^ provided the relevant data regarding the improvement of VAS at 24 months, and it showed that the improvement of VAS was more in BMMNCs group than that of controlled group (MD = 6.87; 95% CI = 1.84–11.89, *P* = .007) (Fig. 7B).

Subgroup analysis was performed according to different therapy dose of BMMNCs. Among them, 3 trials^[32,35,36]^ reported the VAS at 24 months with cell therapy dose unit is 10^8^, and the results showed that the difference of the VAS at 24 months between 2 groups was statistically significant (MD = −12.21; 95% CI = −16.26 to −8.17, *P* < .00001), and only 1 trial^[37]^ reported the VAS at 24 months with cell therapy dose unit is 10^9^, it also showed that the difference of the VAS at 24 months between 2 groups was statistically significant (MD = −9.54; 95% CI = −11.31 to −7.77, *P* < .00001). Subgroup analysis was performed according to the presence or absence of implants. Among them, 3 trials^[35–37]^ reported the VAS at 24 months with implants, and the results showed that the difference of the VAS at 24 months between 2 groups was statistically significant (MD = −9.66; 95% CI = −11.23 to −8.09, *P* < .00001), and 3 trials^[32,43,46]^ reported the VAS at 24 months without implants, it also showed that the difference of the VAS at 24 months between 2 groups was statistically significant (MD = −14.63; 95% CI = −17.21 to −12.06, *P* < .00001) (Table 3).

#### 3.4.2. Other functional score.

**Figure 8.**
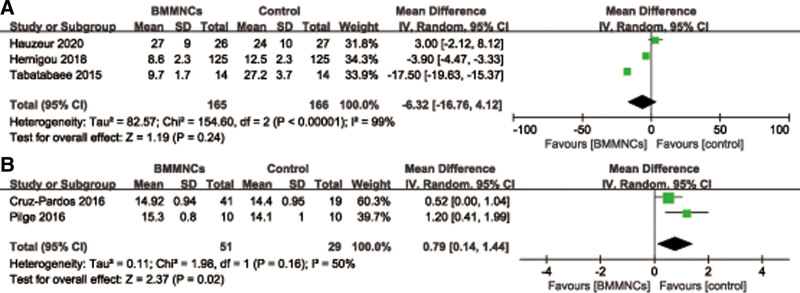
Forest plot of the WOMAC index (A) and Merle D’Aubigne and Postel hip score (B). WOMAC = The Western Ontario and McMaster University Osteoarthritis Index.

Three^[31,32,43]^ of 5 studies with 331 patients provided the relevant data regarding the WOMAC index at last follow-up time. A random effects model was used due to high heterogeneity (*I*^2^ = 99%, *P* < .00001). And pooled data showed that there was no statistically significant difference of the WOMAC index between 2 groups (MD = −6.32; 95% CI = −16.76 to 4.12, *P* = .24) (Fig. 8A). However, the opinions of Gangji et al^[29]^ and Ma et al^[37]^ were that there was a significant reduction in the WOMAC index (*P* = .013; *P* < .001) within the bone-marrow-graft group.

And only 2 trials^[27,41]^ with 80 patients reported the relevant data regarding Merle D’Aubigne and Postel hip score at 24 months. There was statistically significant difference of Merle D’Aubigne and Postel hip score between 2 groups (MD = 0.79; 95% CI = 0.14–1.44, *P* = .02) (Fig. 8B). Four studies described the Lequesne index. Hauzeur et al^[31]^ suggest no differences were found for the Lequesne indexes. The other 3 trials^[28,29,37]^ reported a significant reduction in the Lequesne index.

#### 3.4.3. Radiographic progression rate.

**Figure 9.**
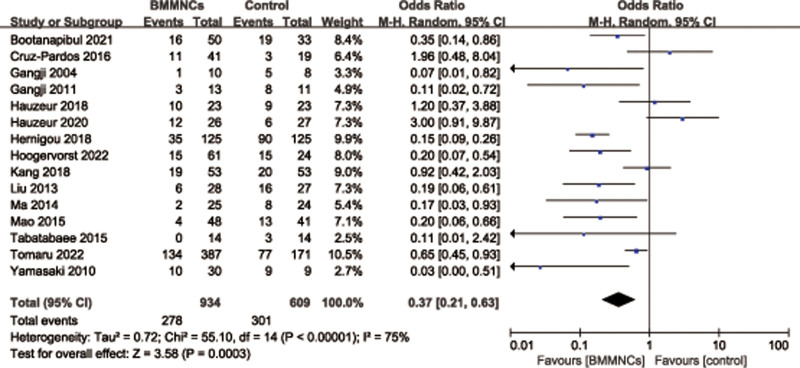
Forest plot of radiographic progression rate.

**Figure 10.**
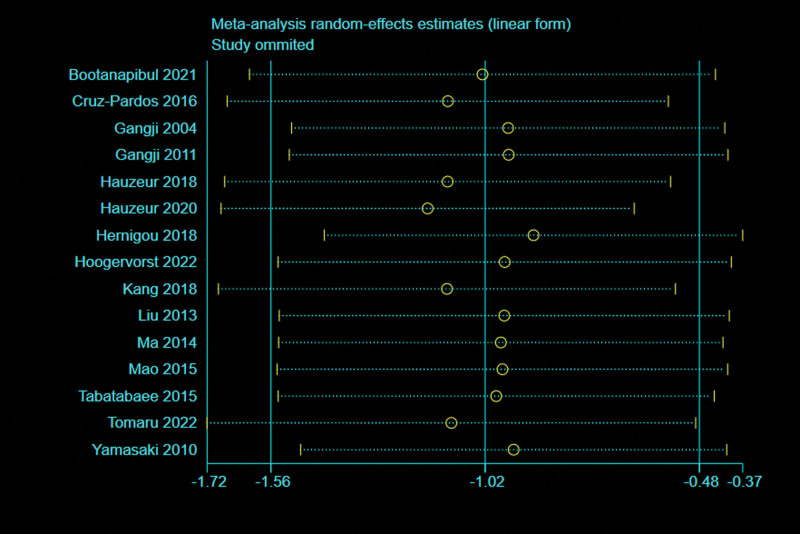
Sensitivity analysis of radiographic progression rate.

A total of 15 studies^[5,13,27–33,35,37,38,43–45]^ with 1543 patients reported the relevant data regarding the radiographic progression rate. There was high heterogeneity observed among articles (*I*^2^ = 75%, *P* < .00001), therefore, a random effects model was used. Pooled Data showed a significantly lower the radiographic progression rate in BMMNCs group compared with control group (OR = 0.37; 95% CI = 0.21–0.63, *P* = .0003) (Fig. 9). Considering the significant heterogeneity, the Stata software was used for further sensitivity analysis (Fig. 10), and the results showed that the total combined effect size of the radiographic progression rate didn’t change significantly after removing single study one by one, suggesting that the results were robust.

Subgroup analysis was performed according to different follow-up periods. Among them, 6 trials^[29,30,35,37,43,45]^ reported the radiographic progression rate with follow-up time at 12 to 24 months, and the results showed that the difference of the radiographic progression rate between 2 groups was statistically significant (OR = 0.20; 95% CI = 0.07–0.61, *P* = .004), and 9 trials^[5,13,27,28,31–33,38,44]^ reported the radiographic progression rate with follow-up time more than 36 months, it also showed that the difference of the radiographic progression rate between 2 groups was statistically significant (OR = 0.46; 95% CI = 0.24–0.88, *P* = .02). Subgroup analysis was performed according to different therapy dose of BMMNCs. Among them, 4 trials^[32,33,35,38]^ reported the radiographic progression rate with cell therapy dose unit is 10^8^, and the results showed that the difference of the radiographic progression rate between 2 groups was statistically significant (OR = 0.27; 95% CI = 0.10–0.71, *P* = .008), and 6 trials^[28–31,37,45]^ reported the radiographic progression rate with cell therapy dose unit is 10^9^, it showed that no statistically significant difference of the radiographic progression rate between 2 groups (OR = 0.30; 95% CI = 0.07–1.25, *P* = .10). Subgroup analysis was performed according to the presence or absence of implants. Among them, 4 trials^[35,37,38,45]^ reported the radiographic progression rate with implants, and the results showed that the difference of the radiographic progression rate between 2 groups was statistically significant (OR = 0.15; 95% CI = 0.07–0.31, *P* < .00001), and 11 trials^[5,13,27–33,43,44]^ reported the radiographic progression rate without implants, it also showed that the difference of the radiographic progression rate between 2 groups was statistically significant (OR = 0.47; 95% CI = 0.25–0.89, *P* = .02) (Table 3).

#### 3.4.4. Complication.

**Figure 11.**
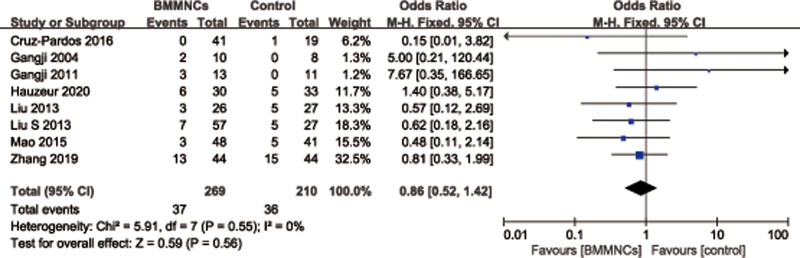
Forest plot of the rate of complication.

Eight studies^[27–29,31,35,36,38,46]^ with 479 patients provided the relevant data regarding the rate of complication. A fixed effects model was used because of no significant heterogeneity (*I*^2^ = 0%, *P* = .55). There was no statistically significant difference of the rate of complication between 2 groups (OR = 0.86; 95% CI = 0.52–1.42, *P* = .56) (Fig. 11).

Subgroup analysis was performed according to different follow-up times. Among them, 3 trials^[29,35,36]^ reported the rate of complication with follow-up time at 12 to 24 months, and the results showed that there was no statistically significant difference of the rate of complication between 2 groups (OR = 0.77; 95% CI = 0.31–1.90, *P* = .57), and 5 trials^[27,28,31,38,46]^ reported the rate of complication with follow-up time more than 36 months, it also showed that no statistically significant difference of the rate of complication between 2 groups (OR = 1.02; 95% CI = 0.53–1.96, *P* = .95). Subgroup analysis was performed according to different therapy dose of BMMNCs. Among them, 3 trials^[35,36,38]^ reported the rate of complication with cell therapy dose unit is 10^8^, and the results showed that no statistically significant difference of the rate of complication between 2 groups (OR = 0.56; 95% CI = 0.25–0.1.26, *P* = .16), and 3 trials^[28,29,31]^ reported the rate of complication with cell therapy dose unit is 10^9^, it also showed that there was no statistically significant difference of the rate of complication between 2 groups (OR = 2.28; 95% CI = 0.78–6.64, *P* = .13). Subgroup analysis was performed according to the presence or absence of implants. Among them, 3 trials^[35,36,38]^ reported the rate of complication with implants, and the results showed that there was no statistically significant difference of the rate of complication between 2 groups (OR = 0.56; 95% CI = 0.25–1.26, *P* = .16), and 5 trials^[27–29,31,46]^ reported the rate of complication without implants, it also showed that no statistically significant difference of the rate of complication between 2 groups (OR = 1.13; 95% CI = 0.59–2.16, *P* = .72) (Table 3).

### 3.5. Publication bias

**Figure 12.**
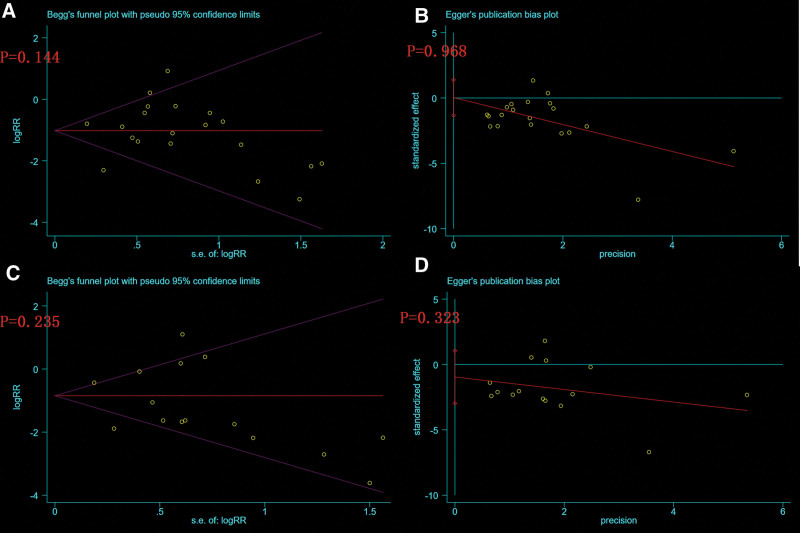
Publication bias plot of Begg’s test and Egger’s test of THA conversion rate and radiographic progression rate. (A) Begg’s test of THA conversion rate (*P* = .144); (B) Egger’s test of THA conversion rate (*P* = .968); (C) Begg’s test of radiographic progression rate (*P* = .235); and (D) Egger’s test of radiographic progression rate (*P* = .323). THA = total hip arthroplasty.

Begg’s test and Egger’s test were used to analyze the publication of several indexes, such as the THA conversion rate and radiographic progression rate. No significant publication bias was found and all *P* values were greater than .05 (Fig. 12).

## 4. Discussion

Although the pathogenesis of ONFH is multifactorial and sometimes poorly understood, it is generally accepted that the variety of traumatic and non-traumatic insults compromise the already precarious circulation of the femoral head, resulting in bone ischemia triggering the death of bone marrow and osteocyte, and eventually collapse of the necrotic segment.^[47]^ Therefore, vascular regeneration and bone and cartilage regeneration are important mechanisms of femoral head necrosis repair. BMMNCs can be isolated from individual patients without heterologous hazards or ethical questions and no direct complications have ever been reported following this type of therapy. Many studies have shown that BMMNCs contain endothelial progenitor cells, osteogenic progenitor cells, mesenchymal stem cells, platelets and other cells.^[19,29]^ These cells show multi-potential capacities to differentiate into endothelial cells, osteocytes, chondrocytes, osteoblasts, adipocytes and so on^[48]^ after being transplanted in the local necrotic area, meanwhile, It can also secrete and release a variety of growth factors (such as VEGF-A), induce regeneration and repair reactions, and enhance the formation of neovascularization in necrotic areas and the regeneration and repair of bone and cartilage tissues.^[49–52]^ Therefore, BMMNCs therapy has a potentially important role in the repair of ONFH.

The results of this study show that BMMNCs therapy can significantly reduce the mid- and long-term rate of THA, improve hip function, including increase Hairrs score and MerleD Aubigne Postel score, decrease Lequesne index of hip joint, reduce the degree of hip pain, delay the progression of imaging stage and reduce the femoral head necrosis volume. In terms of safety, BMMNCs therapy of ONFH does not increase the incidence of complications.

Hernigou et al^[32]^ first proposed the application of BMMNCs combined with core decompression in the treatment of 189 cases of femoral head necrosis. The results showed that the application of mononuclear cells significantly improved the Harris score of hip joint, and believed that the more the number of hip progenitor cells transplanted, the better the prognosis of patients. In addition, multiple previous meta-analyses^[22–24,26,53]^ have found that bone marrow stem cell treatment of femoral head necrosis can improve Hairrs score of hip joint, reduce VAS score, and delay disease progression, which is consistent with the results of this study. Based on the above studies, this study explores the effects of different follow-up times, different cell doses, and with or without implants on hip joint function and hip pain degree. Subgroup analysis was performed according to different follow-up time. The results showed that the Hairrs score of the hip joint was significantly improved at 12 to 24 months and more than 36 months, the WOMAC score was significantly decreased at 24 months of follow-up, and the hip Lequesne index score decreased at 24 months of follow-up after cell therapy, indicating that BMMNCs therapy can improve hip function in the medium and long term. The VAS score decreased during the follow-up of 3 to 6 months, but the difference was not statistically significant. That was significantly lower than the non-cell therapy group at 24 months, indicating that the pain symptoms after cell therapy were not significantly relieved in the early stage, but it could significantly reduce the degree of hip pain in the medium and long term. Different doses of cell therapy have a certain impact on the efficacy. Theoretically, increasing the dose of cell therapy in an appropriate range can increase the number of stem cells, osteoblasts and osteocytes in the area of femoral head necrosis, which may enhance the local repair ability. Most of the cell doses used in the included studies was between 10^8^ and 10^10^. Subgroup analysis was conducted according to the cell therapy dose. Compared with the control group, both the cell therapy doses of 10^8^ and 10^9^ could improve the hip Hairrs score and significantly reduce the VAS pain score. The intervention measures used in the control group in this study were core decompression only and core decompression combined with implants, and the differences in the control group may have a certain impact on the combined results. In order to explore its effect, a subgroup analysis was conducted according to the different intervention measures in the control group. The results showed that BMMNCs combined with core decompression in the treatment of ONFH could improve hip joint function and reduce hip pain regardless of implant.

In previous studies, it is controversial whether BMMNCs can reduce the incidence of THA. Some studies^[5,13,44,53]^ have found that BMMNCs therapy can improve the postoperative hip Hairrs score, delay the stage progression of femoral head necrosis, and reduce the occurrence of THA. However, other studies^[31,54]^ have shown that although BMMNCs therapy can delay hip collapse, there is no statistically significant difference in the incidence of THA between cell therapy and core decompression. Considering the confounding of bone marrow stem cells in the experimental group and the small number of included literature in the previous study, the evidence strength of the results was reduced. This study summarized and improved on the basis of previous studies, only included studies of BMMNCs therapy, and explored the effects of different follow-up times, different cell doses, and with or without implants on the progression of femoral head necrosis and the occurrence of THA. In a study comparing the efficacy of BMMNCs and core decompression published by Hernigou et al,^[32]^ the follow-up time was as long as 30 years, involving 250 hips of stage I and II necrotic femoral head. The results showed that BMMNCs therapy could significantly reduce hip collapse and total hip replacement rate compared with core decompression at 30 years of follow-up. It has been demonstrated that BMMNCs transplantation can reduce the number of revision and re-revision after total hip arthroplasty. However, other studies^[39,55]^ have shown that there is no statistical difference in the incidence of THA between BMMNCs and core decompression therapy at 5 years of follow-up. BMMNCs therapy for femoral head necrosis is controversial in reducing the incidence of THA during long-term follow-up. However, this study found that both the hip replacement rate and the imaging progression rate of cell therapy group were significantly lower than that of non-cell therapy group at 12 to 24 months as well as more than 36 months follow-up, indicating that cell therapy can delay hip collapse and reduce joint replacement rate in the short and medium to long term. The study also found that the dose of mononuclear cells 10^8^ could reduce hip replacement rate and the imaging progression rate, but although there was a trend to reduce the hip replacement rate and the imaging progression rate when the dose of mononuclear cells was 10^9^, the difference between 2 groups was not statistically significant. In the future, more studies on the relationship between the dose of cell therapy and the course of treatment and efficacy are needed. In addition, whether there are implants or not, BMMNCs combined with core decompression in the treatment of femoral head necrosis can delay the progression of imaging, reduce the hip replacement rate, and play a good role in promoting the repair of femoral head necrosis.

There are a few limitations in our study as follows. Firstly, due to the limited availability of RCTs on BMMNCs for the treatment of OFNH, a small number of RCTs were included in this study, the vast majority of the included trials failed to describe detailed information about randomization, allocation concealment, and blinding, as these are the core standards of a well-designed RCT. It is so hard to randomly allocate the patients’ hip joint that most of clinical studies failed to randomize, and some non-RCTs were included. These reasons were contributed to bias of risk of included studies. Secondly, most trials reported positive effects in the BMMNCs for the treatment of ONFH, while negative findings are less likely to be published, implying that publication bias may have existed. Thirdly, it is related to a variety of factors about the progression of OFNH head and the occurrence of THA, there are few studies on different etiologies, stages of necrosis, different cell delivery methods and different courses of treatment in the included literature. And this paper did not conduct in-depth analysis on different etiologies, stages of femoral osteonecrosis, cell therapy dose, different cell delivery methods and different courses of treatment. Of course, this is what we need to study in the future.

## 5. Conclusion

In summary, current evidence supports that BMMNCs therapy could reduce the mid- and long-term rate of THA, improve hip function, alleviated the degree of hip pain, delay the progression of imaging staging and not increase the rate of complication, which maybe serve as a preferred option for treating ONFH. Due to limited quality of the included trials, additional high-quality, well-designed RCTs are required to verify these conclusions in the future.

## Author contributions

**Conceptualization:** Xiao Chen.

**Data curation:** Xiao Chen, Yanji Duan, Chang Chen, Yuan Cao.

**Formal analysis:** Xiao Chen.

**Funding acquisition:** Xiao Chen.

**Investigation:** Xiao Chen.

**Methodology:** Xiao Chen, Jing Chen, Chang Chen.

**Project administration:** Xiao Chen.

**Resources:** Xiao Chen.

**Software:** Xiao Chen, Jing Chen, Yuan Cao.

**Supervision:** Xiao Chen, Jing Chen.

**Validation:** Xiao Chen.

**Visualization:** Xiao Chen.

**Writing – original draft:** Xiao Chen, Jing Chen, Yanji Duan, Chang Chen, Yuan Cao.

**Writing – review & editing:** Xiao Chen, Jing Chen.
